# Variation of Densitometry on Computed Tomography in COPD – Influence of Different Software Tools

**DOI:** 10.1371/journal.pone.0112898

**Published:** 2014-11-11

**Authors:** Mark O. Wielpütz, Diana Bardarova, Oliver Weinheimer, Hans-Ulrich Kauczor, Monika Eichinger, Bertram J. Jobst, Ralf Eberhardt, Marcel Koenigkam-Santos, Michael Puderbach, Claus P. Heussel

**Affiliations:** 1 Department of Diagnostic and Interventional Radiology, University Hospital of Heidelberg, Heidelberg, Germany; 2 Translational Lung Research Center Heidelberg (TLRC), Member of the German Center for Lung Research (DZL), Heidelberg, Germany; 3 Department of Diagnostic and Interventional Radiology with Nuclear Medicine, Thoraxklinik at University of Heidelberg, Heidelberg, Germany; 4 Department of Radiology, German Cancer Research Center (dkfz), Heidelberg, Germany; 5 Department of Pneumology and Respiratory Critical Care Medicine, Thoraxklinik at University of Heidelberg, Heidelberg, Germany; Research Center Borstel, Germany

## Abstract

**Objectives:**

Quantitative multidetector computed tomography (MDCT) as a potential biomarker is increasingly used for severity assessment of emphysema in chronic obstructive pulmonary disease (COPD). Aim of this study was to evaluate the user-independent measurement variability between five different fully-automatic densitometry software tools.

**Material and Methods:**

MDCT and full-body plethysmography incl. forced expiratory volume in 1s and total lung capacity were available for 49 patients with advanced COPD (age = 64±9 years, forced expiratory volume in 1s = 31±6% predicted). Measurement variation regarding lung volume, emphysema volume, emphysema index, and mean lung density was evaluated for two scientific and three commercially available lung densitometry software tools designed to analyze MDCT from different scanner types.

**Results:**

One scientific tool and one commercial tool failed to process most or all datasets, respectively, and were excluded. One scientific and another commercial tool analyzed 49, the remaining commercial tool 30 datasets. Lung volume, emphysema volume, emphysema index and mean lung density were significantly different amongst these three tools (p<0.001). Limits of agreement for lung volume were [−0.195, −0.052l], [−0.305, −0.131l], and [−0.123, −0.052l] with correlation coefficients of r = 1.00 each. Limits of agreement for emphysema index were [−6.2, 2.9%], [−27.0, 16.9%], and [−25.5, 18.8%], with r = 0.79 to 0.98. Correlation of lung volume with total lung capacity was good to excellent (r = 0.77 to 0.91, p<0.001), but segmented lung volume (6.7±1.3 – 6.8±1.3l) were significantly lower than total lung capacity (7.7±1.7l, p<0.001).

**Conclusions:**

Technical incompatibilities hindered evaluation of two of five tools. The remaining three showed significant measurement variation for emphysema, hampering quantitative MDCT as a biomarker in COPD. Follow-up studies should currently use identical software, and standardization efforts should encompass software as well.

## Introduction

Chest multidetector computed tomography (MDCT) remains the gold standard for imaging-based phenotyping of cigarette smoke-induced chronic obstructive pulmonary disease (COPD) [1], [2]. Two main features, related to distinct clinical phenotypes, may be assessed: airway remodeling and emphysema [3]–[7]. Densitometry of the lung parenchyma based on Hounsfield units (HU) is currently the method of choice for non-invasive objective emphysema quantification [1], [8], [9]. As such it has been implemented in various clinical trials including the COPDGene study [10], [11]. Its acceptance in clinical routine is rapidly broadening, leading to an implementation into the workflow of interventional emphysema therapy in many specialized centers [12]. Usually, lung volumes with a density lower than the commonly used threshold of −950 HU are accepted as emphysema [3]. Densitometry of emphysema varies with inspiration depth [13], [14], exposure parameters incl. low-dose scans [15], [16], and reconstruction settings incl. kernel, iterative algorithms vs. filtered back-projection, and slice thickness [17]–[20]. Between different scanner manufacturers variation of lung density and emphysema is thought to be reproducible within close margins using similar scanning and reconstruction parameters [16]. For a large multicenter trial (COPDGene), a dedicated lung phantom was employed to allow for inter-center quality control aimed at standardized examination parameters [10]. A broad standardization of densitometry including repeat calibration of the individual setup at each site encompassing the individual scanner, reconstruction and the post-processing software with dedicated emphysema phantoms is currently missing. However, these steps are essential in the implementation of densitometry as a routine imaging-derived biomarker across different centers, comparable to efforts undertaken for laboratory testing of biomarkers, e.g. for blood samples [21]. In a previous report more than seven years ago, we compared different software tools, which needed user-interaction to complete lung segmentation and implied processing times around 59–105 minutes per patient [22]. Since then, development in software algorithms and computer performance has led to the introduction of several scientific and also commercially available tools, which warrant automatic lung segmentation and densitometry within the work-up of a routine diagnostic chest MDCT scan, delivering emphysema quantification without user-interaction. At present, there is also no clear consensus on which parameters should be measured, and how the respective software algorithms should work in emphysema quantification. This leads to an often confusing usage of different terminology between different software. None of these tools, have been validated against each other, and measurement variation between different software tools is not known. Therefore, the present study was conducted to determine the measurement variation between two commercially available state-of-the-art products and an in-house scientific tool for emphysema quantification based on the identical MDCT examinations of advanced COPD patients suffering from emphysema.

## Materials and Methods

### Ethics Statement

The study was carried out as a retrospective analysis of clinically indicated MDCT performed in July 2012, and has been approved by the Ethics Committee of the Medical Faculty of the University of Heidelberg. Informed written consent for examination and further data processing was obtained from patients or legal guardians.

### Patient Population

49 COPD patients referred to MDCT for the evaluation of endoscopic lung volume reduction procedures were enrolled into the study. Table 1 shows a summary of the patients’ clinical characteristics.

**Table 1:** Patient characteristics.

Number of subjects	49
**Age (years)**	64±9
**Male/female**	24/25
**Pack years**	39±22
**Weight (kg)**	66.2±13.3
**BMI (kg/m^2^)**	23.3±2.6
**GOLD I/II/III/IV**	1/1/26/21
**FEV1 (l/s)**	0.8±0.3
**FEV1 (%)**	31±6
**TLC (l)**	7.7±1.7
**TLC (%)**	128±11
**RV (l)**	5.3±1.4
**RV (%)**	230±31

BMI = body mass index, FEV1 = forced expiratory volume in 1 s, TLC = total lung capacity, RV = residual volume. Percentage values refer to the predicted volumes.

### Pulmonary Function Testing

Whole-body plethysmography (MasterScreen Body, E. Jaeger, Hoechberg, Germany) was performed according to the guidelines of the European Respiratory Society and the standards of the American Thoracic Society (ATS) [23], and the European Coal and Steal Community (ECSC) predicted values served as the standard of reference [24]. The following lung function parameters (absolute and percent predicted values) were used for further analysis: forced expiratory volume in 1s (FEV1), residual volume (RV), and total lung capacity (TLC). To estimate the degree of hyperinflation, the RV to TLC ratio was calculated (RV/TLC).

### Multidetector Computed Tomography

Exclusively non-enhanced thin-section MDCT was routinely performed in supine position as recommended for COPD [4], [25]. Before scanning, all patients received an instructed training to achieve full end-inspiratory breath-hold. All patients were examined with a 4-slice Volume Zoom helical computer tomograph (Siemens Medical Solutions AG, Forchheim, Germany) with a dose-modulated protocol at 120 kV, 70 mAs effective, a collimation of 1.25 mm, and pitch 2 leading to a typical breath-hold length of 19 s. Reconstruction was performed with a slice thickness of 1.25 mm and 1.0 mm increment in a medium soft B40f algorithm as recommended for densitometry [3], [6]. The scale of attenuation coefficients with this system ranges from −1,024 to +3,072 HU. The system was calibrated for water quarterly and after major maintenance, and for air daily. All examinations were visually inspected by a reader with more than 5 years of experience in chest radiology for adequate inspiration, absence of significant motion artifacts and inclusion of all parts of the chest, similar to the inclusion criteria of the COPDGene study [10]. Minor respiratory artifacts were accepted, if they did not impair diagnostic image quality, e.g. slight diaphragmatic motion. The examination protocol and equipment were kept constant during the study period.

### Quantitative MDCT Densitometry Tools

#### YACTA

YACTA (“yet another CT analyzer”) (version 1.1, programming by O. W.) analyzed each stack of around 300 images per patient fully automatically, as employed in previous studies [6], [25]–[27]. YACTA operates in a server-mode and may receive DICOM data directly from the PACS system. Because it is an in-house software, the exact steps of lung and airway segmentation, and emphysema quantification are controlled for and described in detail elsewhere [28], [29]. Neither user interaction nor manual correction of the segmentation were carried out. A lung voxel was assigned to emphysema if its density equaled or was below the threshold of −950 HU [1], [3], with a noise correction for voxels with −910 to −949HU that needed at least 4 adjacent voxels with a density of < −950 HU. The following variables were computed and exported as a structured report and to an in-house scientific data-base for further analysis: lung volume (LV), lung weight, trachea volume, emphysema volume (EV), emphysema surface, emphysema index (EI), mean lung density (MLD), and 15^th^ percentile of lung density histogram. Transfer of the results sheet into the PACS was not available. Measurement results and processing time were recorded for further analysis.

#### LowATT

Aquarius is a commercially available visualization software package (version 4.4.7, TeraRecon, Foster City, California, USA). For emphysema quantification the integrated semi-automated tool LowATT was employed. The MDCT datasets were sent from the PACS to the respective post-processing server. Then each patient was loaded manually into the software surface on a dedicated workstation. A pre-selection of the emphysema threshold is possible, and the interval from −1024 to −950 HU was used for the present study. Results are presented as a color-coded emphysema visualization in multiplanar reformats (MPR) and a results sheet, which may be sent back to the PACS. The following parameters were calculated by lowATT: lung volume (equals LV), low attenuation volume (equals EV) and low attenuation volume in percent (equals EI). Mean lung density was not available. Measurement results and processing time were thus recorded for further analysis.

#### Pulmo 3D

Syngo.Via (version VA20B, Siemens Medical Solutions, Forchheim, Germany) is a commercial post-processing software environment for routine diagnostics. The MDCT datasets were sent from the PACS to the respective post-processing server. Then each patient was loaded manually into the integrated tool Pulmo 3D for densitometry. The emphysema threshold can be selected manually, and −950 HU was chosen as for the other software. Densitometry results are displayed as color-coded emphysema maps in MPR and a results sheet, which may be sent back to the PACS. Parameters measured were: lung volume (equals LV), mean lung density (equals MLD), full width at half maximum of lung density histogram, and low attenuation volume in percent (equals EI). The EV needed to be calculated manually by multiplying low attenuation volume in percent with lung volume. Measurement results and processing time were thus recorded for further analysis.

#### Software excluded from analysis

Initially, we intended to study another free open-source scientific tool as well as a commercial product platform by a large vendor. The open-source tool could interpret 11 from 49 (22%) datasets only, with an unexpected halt during the segmentation process of the remaining 38 datasets. Adequate error management possibilities were not available to the average user. The commercial tool loaded all datasets into the viewer, but halted during segmentation in all datasets. There was no possibility to correct the error by the user. Thus, both tools were excluded from further analysis.

### Statistical Analysis

Computational results were reviewed by a reader with more than 5 years of expertise in chest radiology, preceding their statistical evaluation. All data were recorded in a dedicated database (Excel, Microsoft Corp., Redmond, USA) and analyzed using SigmaPlot (Systat Software GmbH, Erkrath, Germany) software. Parametric data are displayed as mean ± standard deviation, non-parametric data as median ± median average deviation. Measurement results with similar meaning provided all three software tools are segmented LV (l), segmented EV (l) and EI (%), which were used for statistical comparison by repeated measures analysis of variance (RM ANOVA) (LV, EV) or RM ANOVA on ranks (EI) with post-hoc tests as appropriate. MLD in HU was computed by YACTA and Pulmo 3D only, and compared by Wilcoxon signed rank test. In a tandem analysis differences were plotted against the mean of two methods by using the approach described by Bland and Altman [30]. We also calculated coefficients of variation as the ratio of the standard deviation of the mean inter-software difference to the mean difference of both measurements. LV, EV and EI were correlated with lung function tests, and Pearson correlation coefficient or Spearman rank order correlation coefficient r were computed as appropriate. A p-value of<0.05, corrected with the Bonferroni-Holm method in case of multiple comparisons, was considered statistically significant [31].

## Results

### Data processing

**Figure 1:**
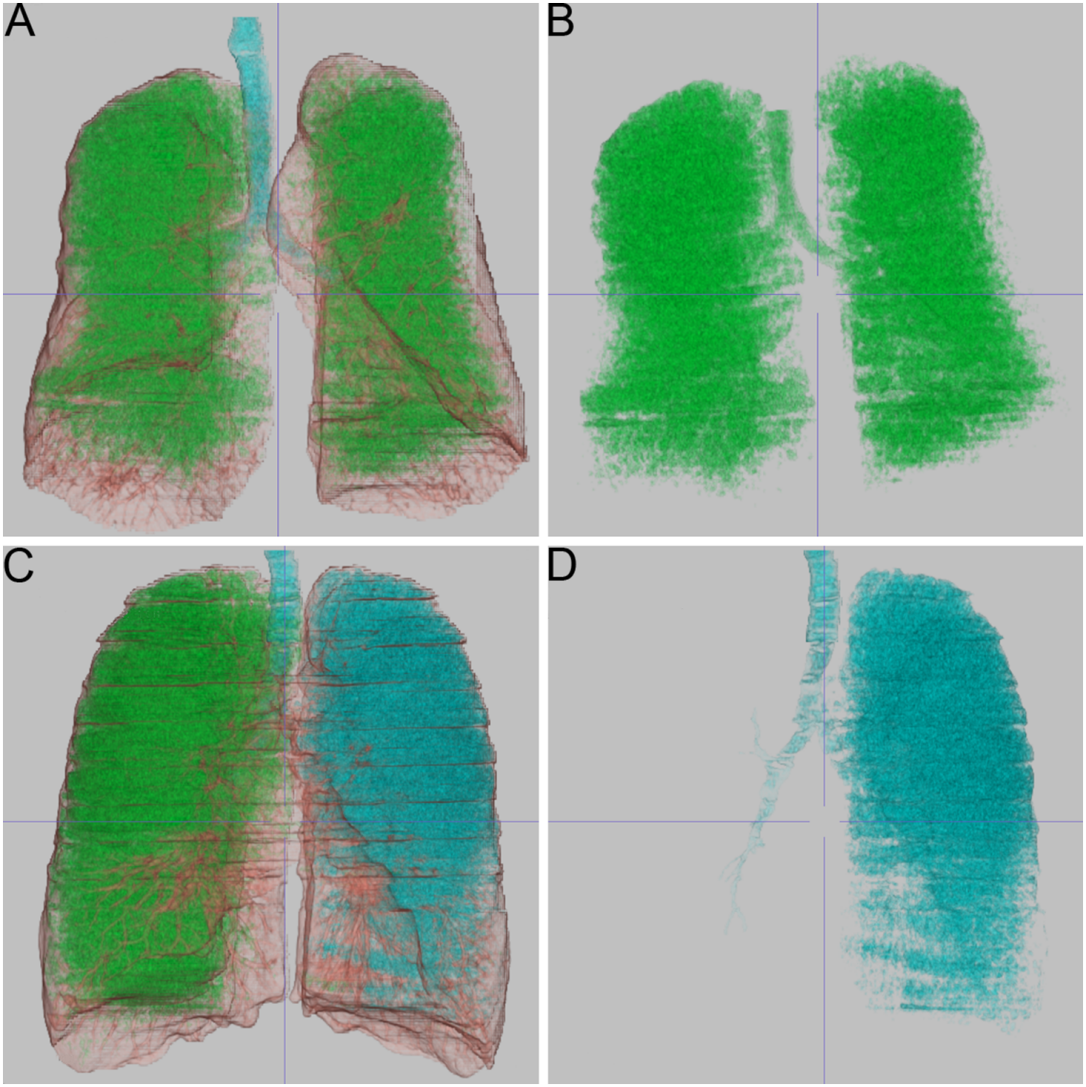
Emphysema visualization by density maps. Every software offered volume-rendering of segmented central airways (highlighted in blue), lung (brown) and emphysema (green). Leakage of the segmentation algorithms from airways into the parenchyma and vice versa are frequent sources of error in densitometry on computed tomography datasets, which results in faulty results or unexpected halt of the software tool. A, B 73–year-old patient, FEV1 = 43%, emphysema index calculated with 37% and 40%, not processed by the third software tool. A suggests correct segmentation of airways and emphysema, whereas isolated display of emphysema in B demonstrates that the software has assigned the airway tree actually also to the emphysema volume. C, D 52–year-old patient, FEV1 = 20%, emphysema index calculated with 41% and 45%, not processed by the third software tool. Visualization of the segmented airways and emphysema in C, and selective display of the segmented airways in D show that airway segmentation leaked into the left lung. Respiratory artifacts can be appreciated in C, which have obscured the airway wall of segmental airways on the left side.

All 49 (100%) datasets were evaluated by YACTA without user interaction within a runtime of around 3 minutes per patient, depending on the amount of emphysema. No obvious segmentation errors regarding airways or lung volume were observed upon inspection of the results in the three-dimensional MPR mode. LowATT completely evaluated all 49 (100%) datasets also, without any unexpected halt during data processing. Mean runtime was below 3 minutes per patient from sending the patient to the server to completion of the results. A review of the segmentation results by color-coded MPR revealed, that in 17 cases (35%) central airways were segmented as emphysema; in a single case the left lung was segmented as belonging to the airway tree (Figure 1). These cases first remained in the analysis, because the intention of our study was an user-independent approach. Pulmo 3D could process 30 of the 49 (61%) datasets loaded into the viewer in less than 3 minutes, but failed to generate results for the remaining 19 datasets, without providing a specific error protocol or management. The 30 datasets processed were included into the analysis and did not show obvious errors in airways or lung segmentation upon inspection of the color-coded MPR. In 9 out of 17 cases in which lowATT delivered erroneous results segmenting central airways as emphysema, Pulmo 3D did not process the dataset at all. Only 21 (43%) datasets altogether were processed without obvious segmentation errors by all of the three software tools.

### Variation of lung volume, emphysema volume and emphysema index

**Figure 2:**
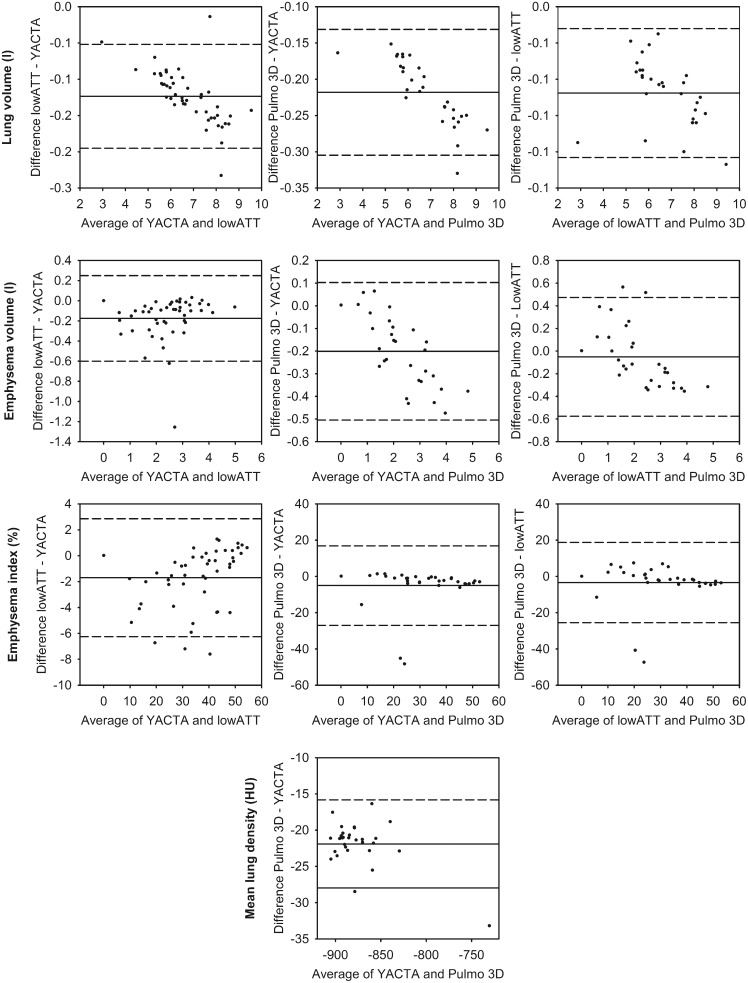
Variation of densitometry. Bland-Altman-plots are given for each inter-software comparison for lung volume, emphysema volume, emphysema index and mean lung density. The central line indicates the mean difference and the dashed lines indicate upper and lower limits of agreement.

To test whether the software tools deliver similar measurements, we compared the output variables LV, EV and EI. The means of these were significantly different amongst all three tools (p<0.001) (Table 2). Figure 2 and Table 3 summarize the results of a tandem comparison of the three tools. For LV, the largest differences were seen for Pulmo 3D vs. YACTA with a mean difference of −0.218 l and limits of agreement as wide as −0.305 to −0.131 l. For EV, Pulmo 3D vs. lowATT had a low mean difference of −0.051 l, but the widest limits of agreement from −0.575 to 0.473 l. In the case of EI, which is computed from LV and EV, differences ranged from −5.0 to −1.7%. The limits of agreement were max. −25.5 to 18.8% again for Pulmo 3D vs. lowATT. MLD could be compared for Pulmo 3D vs. YACTA only, delivering a difference of −21 HU and limits of agreement from −28 to −16 HU (Table 3).

**Table 2:** Overview of the densitometry results.

	YACTA	lowATT	Pulmo 3D	p
**LV (l)**	6.824±1.255	6.657±1.251	6.689±1.356	<0.001
**EV (l)**	2.514±0.991	2.339±1.025	2.195±1.043	<0.001
**EI (%)**	38.8±8.8	37.0±9.4	33.5±9.5	<0.001
**MLD (HU)**	−877±8		−895±13	<0.001

LV = lung volume, EV = emphysema volume, EI = emphysema index, MLD = mean lung density, HU = Hounsfield units.

**Table 3:** Variation of densitometry.

		lowATT - YACTA	Pulmo 3D - YACTA	Pulmo 3D - lowATT	p-value
**LV (l)**	**r**	1.00	1.00	1.00	<0.001
	**ΔLV**	−0.124	−0.218	−0.088	<0.001
	**Limits of agreement**	−0.195, −0.052	−0.305, −0.131	−0.123; −0.052	
	**Coefficient of variation**	0.3	0.2	0.2	
**EV (l)**	**r**	0.98	0.99	0.98	<0.001
	**ΔEV**	−0.175	−0.201	−0.051	<0.001
	**Limits of agreement**	−0.600, 0.250	−0.505, 0.103	−0.575, 0.473	
	**Coefficient of variation**	1.2	0.8	5.2	
**EI (%)**	**r**	0.98	0.79	0.80	<0.001
	**ΔEI**	−1.7	−5.0	−3.4	<0.05
	**Limits of agreement**	−6.2, 2.9	−27.0, 16.9	−25.5, 18.8	
	**Coefficient of variation**	1.4	2.2	3.3	
**MLD (HU)**	**r**		0.99		<0.001
	**ΔMLD**		−21		<0.001
	**Limits of agreement**		−28, −16		
	**Coefficient of variation**		0.1		

LV = lung volume, EV = emphysema volume, EI = emphysema index, MLD = mean lung density, HU = Hounsfield units. Differences (Δ) and limits of agreement were calculated in accordance with the approach of Bland and Altman.

We repeated the comparison of the measurement results with the remaining 21 datasets after removal of those with obvious segmentation errors (Tables S1 and S2). Importantly, even after this manual interaction densitometry results for LV, EV, EI and MLD remained significantly different between the three tools (Table S1). The mean differences, limits of agreement and coefficients of variation between the software tools for LV, EV and MLD were not changed substantially. Only regarding EI, differences were now lower than 2%, and limits of agreement did not exceed ±8% (Table S2).

### Correlation with lung function testing

**Figure 3:**
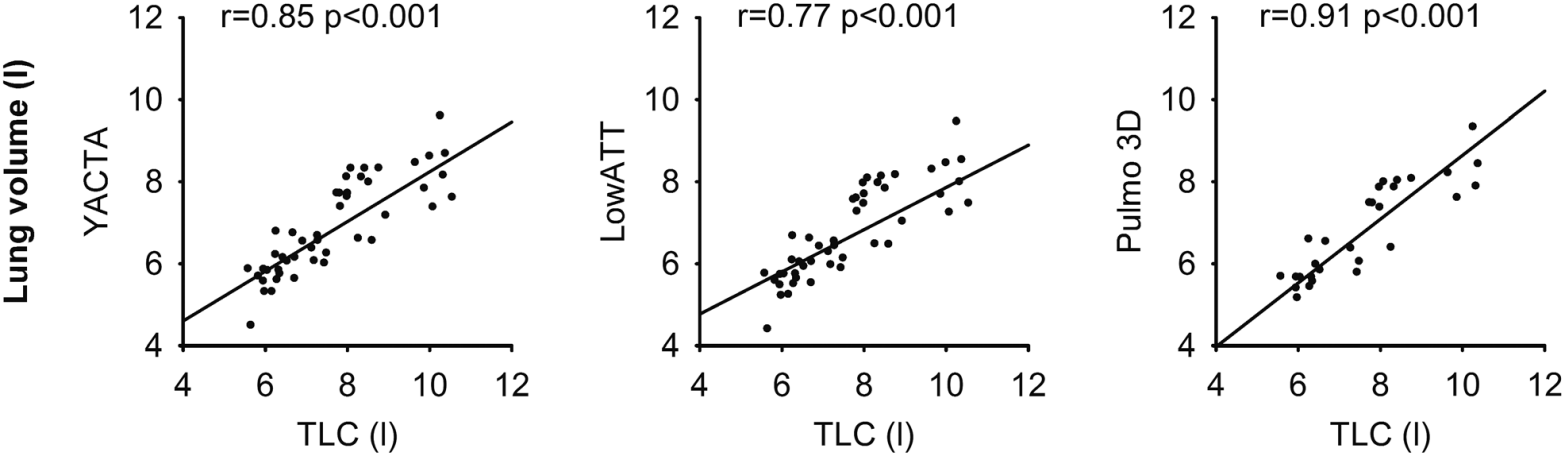
Correlation with lung function testing. The segmented lung volume provided by each individual software showed a good correlation with the total lung capacity (TLC) as measured by lung function testing. Pearson correlation coefficient r and respective p-value are indicated for each plot.

A previous study in patients with COPD suggested that plethysmography may overestimate TLC in obstructive lung disease [32]. In the absence of another standard of reference, we compared segmented lung volumes derived from the three softwares to TLC measured by lung function testing. Usually, this is done to validate whether MDCT was performed at full inspiratory breath hold. We found that segmented LV (6.7±1.3–6.8±1.3 l) were significantly lower than TLC (7.7±1.7 l) (p<0.001) as expected (Table 1 and 2). Correlation of lung volumes with TLC was good to excellent for all three softwares, and the correlation coefficient was highest for Pulmo 3D (r = 0.91) (Figure 3, Table S3). However, there was no relevant correlation of EV or EI with other lung function parameters (Table S3 and S4).

## Discussion

In order to introduce quantitative MDCT into routine patient work-up in emphysema and COPD care, it is necessary to agree on and strictly control for examination protocols, post-processing, measurement parameters and parameter interpretation [21]. These parameters must thus have a high reproducibility among different sites covering different CT scanners and software equipment. The present study sought to investigate the measurement variability of densitometry among different software tools on a predefined set of thin-section MDCT from COPD patients. Measurement results for segmented lung volumes, emphysema volumes and, consecutively, emphysema index were significantly different between one in-house scientific tool and two up-to-date commercially available tools from major vendors. Not all tools were able to process the standard DICOM datasets, even commercially available tools failed to process 39–100% of scheduled data. Only in-house scientific software (YACTA) and a single commercial tool (lowATT) analyzed all data successfully.

In 1979 Harris proposed that the desirable imprecision of two laboratory tests assessing the same parameter should be equal or less than half of the intra-individual biological variation [33]. Reference normal values for emphysema have not been established [34]. The true intra-individual variability of emphysema on quantitative densitometry of repeated MDCT examinations has little been studied. Shaker and colleagues reported a short-term coefficient of variation of 6.8% for emphysema volume with a threshold of −910 HU for repeated examination with the identical scanner and post-processing software in a 2-week interval [35]. For repeated low-dose scans in a 3-month interval Gietema and colleagues found coefficients of variation for the emphysema score (equals EI in our study) of 34% with a limits of agreement (95% confidence interval) from −13.4 to 12.6% at −910 HU, and 58% with a limits of agreement from −1.3 to 1.1% at −950 HU [36]. Hence, the short-term inter-scan intra-individual variability at −950 HU is low. Soejima et al. reported an annual change of relative low attenuation areas (equals EI in our study) between 0.7% and 2.3% (95% confidence interval) in 47 current or former smokers with a threshold of −912 HU [37]. In a more recent study Hoesein et al. reported a mean annual increase of emphysema of 1.07% (confidence interval 1.06–1.09%) in 3,670 former and current smokers at −950 HU [38]. Interestingly, the reported long-term data showed a variability that is within the limits of reported short-term variability. Hence, a clear definition of a tolerable variance in emphysema quantification cannot be given. A more practical approach would be oriented at the clinical consequences of measurement variability. Following Harris’ proposal and considering the previously published data, variability of the EI measured with two different software tools in the present study should be approximately less than 1%. However, the inter-software variability in our study is much higher than the recent reports on emphysema progression with median differences from −5.0 to −1.7% and limits of agreement as wide as −25.5 to 18.8% for EI (Table 3). As currently, there is insufficient data available on the impact of MDCT-derived quantitative parameters on treatment decisions, we are currently unable to give exact margins of tolerable inter-software differences. It is however conceivable, that a measurement variability beyond approx. 10% is relevant for identifying patients suitable for lung volume reduction strategies considering the selection criteria of the VENT Study for example [12]. For this study threshold values for the emphysema index for performing therapy have been postulated, but our results show that these threshold values must be defined for each software used for quantification separately. The inter-software variation may otherwise lead to erroneous exclusion or inclusion for therapy.

Potential sources of error are the steps of lung segmentation, airway segmentation and subsequent emphysema segmentation. A frequently observed problem is the “leakage” of mostly region growing-based algorithms from airways into lung parenchyma, or from segmented emphysema into the airway tree (Figure 1). This leads to substantial miscalculations of the respective volumes, and warrants a validation step by a radiologist before the results are used clinically. Apparently, lowATT provided segmentation results with errors in separating airways from emphysema in some cases. These were not observed with YACTA and Pulmo 3D, but the latter did not provide results for 19 cases at all, some of which showed errors with lowATT also. In total, only 21 datasets could be processed by all three software tools without major segmentation errors. Even in this reduced set of MDCT exams, measurement variability was similar compared to the full patient population. Maximal limits of agreement still were as wide as −7.9 to 7.4% for EI (Table S2). More minor variations in segmentation and noise correction between software are very likely and a source of different densitometry results. A more subtle reason for measurement variation is the extent of airway segmentation into the periphery of the airway tree. Currently, there is no consensus on to which airway generation the airway tree needs to be segmented in order to exclude these airways from lung parenchyma, and thus emphysema. The scope of this study was to evaluate the softwares’ potential for fully-automatic lung densitometry, meaning that neither user-interaction nor correction of the results would be necessary. Moreover, only YACTA provided a tool for manual correction of the computational segmentation results with the software version evaluated for this study.

Similar studies in other important fields of quantitative MDCT have brought up results similar to our study: For example, our study compares well to research by de Hoop et al. who compared six different software tools for automated lung nodule volumetry. The authors found a variation between 16.4–22.3%, which they concluded were unacceptably large with regard to therapy decision in serial exams [39]. A subsequent study by this group revealed similar results [40]. Oberoi et al. investigated the inter-software variation of non-calcified coronary artery plaque quantification. They concluded that inter-platform reproducibility was poor and that serial studies need to use identical software in a research setting [41].

Some limitations of our study need to be addressed. In the absence of a standard of reference in vivo, it is impossible to validate the true EV and thus EI. TLC measured by plethysmography may also be inappropriate as a reference for MDCT-derived LV in the setting of severe COPD [32]. Furthermore, acquisition conditions for plethysmography and MDCT are completely different (prone position, trained technician giving prompt instructions etc.), probably leading to lower segmented LV than TLC also in our study. Thus, we may not evaluate accuracy of the different software tools. The fact that there was no apparent correlation between EV or EI with FEV1, RV or RV/TLC (Table S3 and S4) in contrast to previously published results should not confuse the reader [3], [6]. This is explained by the selection of our patient cohort, mainly consisting of end-stage COPD patients. Thus, there is little variation in lung function impairment and densitometry results, which diminished statistical correlation analysis. It is problematic that two of the tools initially investigated for this study could not process most of the datasets provided although their intended use is vendor-independent. This still may be explained as a conflict of different platforms from different vendors. Furthermore, the examinations were performed on a relatively old 4-slice MDCT system. Other densitometry parameters of emphysema currently subject to debate such as the 15^th^ percentile of the lung histogram were not delivered by the commercial software tools and could not be evaluated in this study [42], [43]. The fact that we compared three out of many other scientific and medical class product tools does not pose a limitation. It is conceivable, that inter-software variability for other tools will range within the same order of magnitude. The in-house scientific software YACTA has not been certified as a medical class product, and may thus not be used in clinical routine.

Following our results we conclude that inter-software variation of densitometry is greater than the natural intra-individual variability of emphysema and beyond acceptable margins for longitudinal studies and identifying patients for lung volume reduction procedures, hampering its broad introduction as a reproducible biomarker. Computational results need to be validated by an experienced radiologist to rule out obvious sources of error. As a perspective, efforts currently undertaken to standardize scanning parameters and quality checks with dedicated attenuation phantoms such as used for the COPDGene study [44] should encompass densitometry software also, to control for all possible factors along the measurement setup from acquisition to computational measurements. Measuring reference emphysema phantoms regularly at each site, similar to quality assurance in laboratories, would foster the acceptance of densitometry as an endpoint in interventional trials, and potentially in clinical routine in the future. Until then, longitudinal studies should be performed using the identical software.

## Supporting Information

Table S1 Results of densitometry after user interaction. N = 21, LV = lung volume, EV = emphysema volume, EI = emphysema index, MLD = mean lung density, HU = Hounsfield units.
(DOCX)

Table S2 Variation of densitometry after user interaction. N = 21, LV = lung volume, EV = emphysema volume, EI = emphysema index, MLD = mean lung density, HU = Hounsfield units. Mean differences (Δ) and limits of agreement were calculated in accordance with the approach of Bland and Altman.
(DOCX)

Table S3 Correlation of quantitative MDCT with lung function. Correlation coefficients were calculated for lung volume (LV), emphysema volume (EV), emphysema index (EI), and mean lung density (MLD) with with forced expiratory volume within 1 s (FEV1, FEV1%), vital capacity (VC), Tiffeneau index (FEV1/VC), residual volume (RV), total lung capacity (TLC), and RV/TLC ratio. *p<0.05.
(DOCX)

Table S4 Correlation of quantitative MDCT with lung function after user interaction. Correlation coefficients were calculated for lung volume (LV), emphysema volume (EV), emphysema index (EI), and mean lung density (MLD) with with forced expiratory volume within 1 s (FEV1, FEV1%), vital capacity (VC), Tiffeneau index (FEV1/VC), residual volume (RV), total lung capacity (TLC), and RV/TLC ratio. *p<0.05.
(DOCX)
