# Supplementary material for: Variation of Densitometry on Computed Tomography in COPD – Influence of Different Software Tools
Source: PLoS One. 2014 Nov 11;9(11):e112898. doi: 10.1371/journal.pone.0112898 (PMC4227864; doi:10.1371/journal.pone.0112898)
Supplement: Table S1 — Results of densitometry after user interaction. N = 21, LV = lung volume, EV = emphysema volume, EI = emphysema index, MLD = mean lung density, HU = Hounsfield units. (DOCX) [file pone.0112898.s001.docx]

|  | **YACTA** | **lowATT** | **Pulmo 3D** | **p** |
| --- | --- | --- | --- | --- |
| **LV (l)** | 6.694±1.470 | 6.571±1.440 | 6.486±1.433 | <0.001 |
| **EV (l)** | 2.329±1.111 | 2.175±1.153 | 2.140±1.020 | <0.001 |
| **EI (%)** | 33.91±8.08 | 30.34±9.52 | 32.80±8.80 | <0.01 |
| **MLD (HU)** | -875±8 |  | -896±8 | <0.001 |
