# Supplementary material for: Variation of Densitometry on Computed Tomography in COPD – Influence of Different Software Tools
Source: PLoS One. 2014 Nov 11;9(11):e112898. doi: 10.1371/journal.pone.0112898 (PMC4227864; doi:10.1371/journal.pone.0112898)
Supplement: Table S2 — Variation of densitometry after user interaction. N = 21, LV = lung volume, EV = emphysema volume, EI = emphysema index, MLD = mean lung density, HU = Hounsfield units. Mean differences (Δ) and limits of agreement were calculated in accordance with the approach of Bland and Altman. (DOCX) [file pone.0112898.s002.docx]

|  |  | **lowATT - YACTA** | **Pulmo 3D - YACTA** | **Pulmo 3D - lowATT** | **p-value** |
| --- | --- | --- | --- | --- | --- |
| **LV (l)** | **r** | 1.00 | 1.00 | 1.00 | <0.001 |
|  | **ΔLV** | -0.123 | -0.209 | -0.085 |  |
|  | **Limits of agreement** | -0.190, -0.057 | -0.292, -0.125 | -0.124, -0.047 |  |
|  | **Coefficient of variation** | 0.3 | 0.2 | 0.2 |  |
| **EV (l)** | **r** | 0.99 | 1.00 | 0.98 | <0.001 |
|  | **ΔEV** | -0.155 | -0.190 | -0.036 |  |
|  | **Limits of agreement** | -0.512, 0.202 | -0.455, 0.074 | -0.575, 0.504 |  |
|  | **Coefficient of variation** | 1.1 | 0.7 | 7.6 |  |
| **EI (%)** | **r** | 0.98 | 0.98 | 0.95 | <0.001 |
|  | **ΔEI** | -1.6 | -1.9 | -0.2 |  |
|  | **Limits of agreement** | -6.7, 3.4 | -5.3, 1.6 | -7.9, 7.4 |  |
|  | **Coefficient of variation** | 1.6 | 0.9 | 19.5 |  |
| **MLD (HU)** | **r** |  | 0.99 |  | <0.001 |
|  | **ΔMLD** |  | -22 |  |  |
|  | **Limits of agreement** |  | -28, -16 |  |  |
|  | **Coefficient of variation** |  | 0.1 |  |  |
