# Supplementary material for: Variation of Densitometry on Computed Tomography in COPD – Influence of Different Software Tools
Source: PLoS One. 2014 Nov 11;9(11):e112898. doi: 10.1371/journal.pone.0112898 (PMC4227864; doi:10.1371/journal.pone.0112898)
Supplement: Table S3 — Correlation of quantitative MDCT with lung function. Correlation coefficients were calculated for lung volume (LV), emphysema volume (EV), emphysema index (EI), and mean lung density (MLD) with with forced expiratory volume within 1 s (FEV1, FEV1%), vital capacity (VC), Tiffeneau index (FEV1/VC), residual volume (RV), total lung capacity (TLC), and RV/TLC ratio. *p<0.05. (DOCX) [file pone.0112898.s003.docx]

|  |  | **FEV1** | **FEV1%** | **RV** | **TLC** | **RV/TLC** |
| --- | --- | --- | --- | --- | --- | --- |
| **YACTA** | LV | 0.32* | -0.23 | 0.68* | 0.85* | 0.02 |
|  | EV | 0.04 | -0.18 | 0.34* | 0.51* | -0.01 |
|  | EI | -0.21 | -0.16 | 0.04 | 0.12 | 0.00 |
|  | MLD | 0.23 | 0.06 | 0.08 | 0.02 | 0.01 |
| **lowATT** | LV | 0.37* | -0.17 | 0.69* | 0.77* | 0.03 |
|  | EV | 0.07 | -0.13 | 0.31* | 0.41* | -0.02 |
|  | EI | -0.15 | -0.13 | 0.05 | 0.12 | -0.02 |
| **Pulmo 3D** | LV | 0.26 | -0.24 | 0.74* | 0.91* | 0.10 |
|  | EV | 0.08 | -0.15 | 0.32 | 0.51* | -0.03 |
|  | EI | -0.11 | -0.10 | 0.05 | 0.26 | -0.25 |
|  | MLD | 0.10 | 0.07 | 0.10 | -0.05 | 0.10 |
